# The association between early MRI and length of disability in acute lower back pain: a systematic review and narrative synthesis

**DOI:** 10.1186/s12891-021-04863-9

**Published:** 2021-11-24

**Authors:** Bara A. Shraim, Muath A. Shraim, Ayman R. Ibrahim, Mohamed E. Elgamal, Basem Al-Omari, Mujahed Shraim

**Affiliations:** 1 College of Medicine, QU Health, Qatar University, Doha, Qatar; 2 NHMRC Centre of Clinical Research Excellence in Spinal Pain, Injury & Health, School of Health & Rehabilitation Sciences, The University of Queensland, Brisbane, QLD 4072 Australia; 3 College of Medicine and Health Sciences, Khalifa University, Abu Dhabi, United Arab Emirates; 4 KU Research and Data Intelligence Support Center (RDISC) AW 8474000331, Khalifa University of Science and Technology, Abu Dhabi, United Arab Emirates; 5 Department of Public Health, College of Health Sciences, Qatar University, QU Health, Doha, Qatar

**Keywords:** Systematic review, Magnetic resonance imaging, Low back pain, Sick leave, Work disability, Return to work

## Abstract

**Background:**

Clinical guideline recommendations are against early magnetic resonance imaging (eMRI) within the first 4 to 6 weeks of conservative management of acute low back pain (LBP) without “clinical suspicion” of serious underlying conditions (red flags). There is some limited evidence that a significant proportion of patients with LBP receive eMRI non- indicated by clinical guidelines, which could be associated with increased length of disability (LOD). The aim of this systematic review was to investigate whether eMRI for acute LBP without red flags is associated with increased LOD. The LOD was defined as the number of disability days (absence from work).

**Methods:**

Medline, EMBASE, and CINAHL bibliographic databases were searched from inception until June 5, 2021. Two reviewers independently assessed the methodological quality of included studies using the Newcastle–Ottawa scale and extracted data for the review. The search identified 324 records, in which seven studies met the inclusion criteria. Three of the included studies used the same study population. Owing to between-study heterogeneity, a narrative synthesis of results was used.

**Results:**

All included studies were of good methodological quality and consistently reported that patients with acute LBP without red flags who received eMRI had increased LOD compared to those who did not receive eMRI. Three retrospective cohort studies reported that the eMRI groups had a higher mean LOD than the no eMRI groups ranging from 9.4 days (95% CI 8.5, 10.2) to 13.7 days (95% CI 13.0, 14.5) at the end of 1-year follow-up period. The remaining studies reported that the eMRI groups had a higher hazard ratio of work disability ranging between 1.75 (95% CI 1.23, 2.50) and 3.57 (95% CI 2.33, 5.56) as compared to the no eMRI groups.

**Conclusion:**

eMRI is associated with increased LOD in patients with acute LBP without red flags. Identifying reasons for performing non-indicated eMRI and addressing them with quality improvement interventions may improve adherence to clinical guidelines and improve disability outcomes among patients with LBP.

**Supplementary Information:**

The online version contains supplementary material available at 10.1186/s12891-021-04863-9.

## Background

Low back pain (LBP) is ranked first globally for years lived with disability among all diseases with an estimated age-standardized point prevalence in 2017 at 7.5% [1]. Further, costs of managing for LBP are very high, exceeding $100 billion per year in the US, and are still increasing [2, 3]. A wide range of complex inter-related factors are associated with an increased length of disability (LOD) among individuals presenting with acute LBP. These include individual factors (e.g., age and gender) [4], occupational factors (e.g., job tenure, physical demand of job, workplace support) [5], regional factors (e.g., workers’ compensation policies [6], socioeconomic factors [7]), and healthcare-related factors (e.g., early opioid prescribing within 15 days of LBP onset [8], early magnetic resonance imaging (eMRI) within the first 4–6 weeks of LBP onset) [9, 10]. In this review, LOD was defined as the number of disability days (absence from work) due to the current episode of LBP [7, 11–13].

It is commonly observed that MRI findings of age-related degenerative changes are prevalent in people without LBP [14, 15]. In addition, a recent study found no relationship between MRI changes in the lumbar spine and pain intensity, health-related quality of life, and depressive and anxiety symptoms among patients with LBP [16]. Furthermore, a systematic review and meta-analysis of imaging strategies for LBP showed that lumbar imaging does not improve clinical outcomes in acute LBP cases without suspected serious underlying conditions [17].

Clinical guidelines for the management of acute nonspecific LBP recommend that imaging, specifically MRI, should not be performed in the first month of conservative management unless red flags (e.g., fracture, tumor, infection, and neurological deficit) are suspected [18–21]. Despite this, eMRI scanning for patients with acute LBP is common (27.7%; 95% confidence interval (CI) 21.3, 35.1) [22] and was found to be associated with increased LOD, more healthcare utilization, and higher medical costs [10–12]. For instance, Mahmud et al., found that eMRI was associated with increased LOD by 102 days (unadjusted 115 vs. 13 days in eMRI and no eMRI groups, respectively) [12], whereas Graves et al., reported that eMRI was associated with an unadjusted 120-day increase in LOD [10]. Undertaking eMRI has been hypothesized to lead healthcare providers to overinterpret the findings and carry out additional and possibly unnecessary interventions, such as surgery, epidural steroid injections physiotherapy, osteopathy, and hospital admission [23–25] and thus lead to an increased LOD [12].

With multiple studies showing an independent association between eMRI and increased LOD among patients with acute LBP, it becomes necessary to synthesize the evidence from those studies. To our knowledge, only one systematic review has assessed the relationship between imaging, including MRI, and absence from work in patients with acute LBP [25]. However, that systematic review did not employ a specific timing for MRI scanning for LBP and included only two studies examining the relationship between eMRI and LOD in LBP cases and synthesized the findings using unadjusted LOD estimates between the eMRI and MRI groups. We are aware of more than two studies reporting on this relationship. Therefore, the aim of this systematic review is to summarize the findings of epidemiologic studies examining the relationship between eMRI and LOD in patients with acute LBP without “clinical suspicion” of serious underlying conditions (hereafter referred to as red flags).

## Materials and methods

### Search strategy

The protocol for this review was registered with the International Prospective Register of Systematic Reviews (PROSPERO) under registration number CRD42021259296 (available from https://www.crd.york.ac.uk/prospero/display_record.php? RecordID = 259,296). Reporting of this systematic review was guided by the Preferred Reporting Items for Systematic Reviews and Meta-Analysis (PRISMA) statement (Supplemental file S1) [26]. We searched Medline, EMBASE, and CINAHL bibliographic databases from their inception until June 5, 2021, using medical subject heading (MeSH) or Emtree and free-text terms on LBP, MRI, and work disability (Supplemental file S2). In addition, reference lists of all relevant papers were searched, and citations of included studies were tracked using the Web of Science Citation Index. No restrictions on language, study design, or time of publication were applied.

### Criteria for considering studies for the review

#### Types of studies

All epidemiologic study designs examining the association between eMRI and LOD in patients with acute LBP were considered for inclusion.

#### Types of participants

Patients with a medical diagnosis of acute LBP, occupational LBP or non-specific LBP were included. Studies including patients with chronic or complicated LBP (e.g., severe injuries, multiple traumas, infection, autoimmune disease, or cancer) were not considered for inclusion in the review.

#### Types of exposures

The exposure was eMRI defined as an MRI of the lumbar spine for LBP within the first 4 to 6 weeks of the first recorded medical visit for the current LBP episode.

#### Types of outcome measures

The main outcome was the measure of association between eMRI and LOD whether it was reported as odds ratios, relative risk, or mean difference in LOD between the eMRI group and the no eMRI group. The LOD was defined as the number of disability days (absence from work) due to the current episode of LBP [7, 11–13].

#### Study selection process

**Fig. 1:**
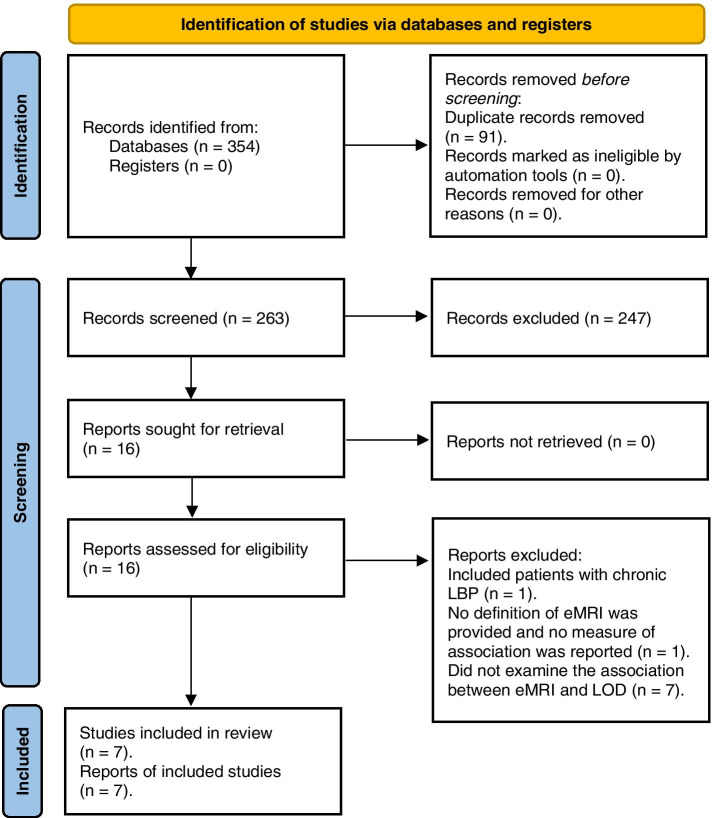
The PRISMA flow diagram of studies in the review

All retrieved records were imported to Covidence web-based application and duplicate records were removed. Initially, titles and abstracts of all records were screened, then full text of relevant papers were reviewed for eligibility for inclusion in the review. The study selection process was conducted independently by two reviewers, and any disagreements were resolved by discussion with a third reviewer. The reasons for study exclusions made during the second stage were reported in the PRISMA flow diagram (Fig. 1).

#### Quality assessment

Methodological quality assessment of included studies was conducted independently by two reviewers using the Newcastle–Ottawa scale for cohort studies (Supplemental file 3) [27] and any disagreements were resolved by discussion with a third reviewer. Where there was a conflict of interest or potential reviewer bias, the reviewer in question was not involved in the quality assessment. This tool assesses the quality of the sample selection process, comparability of cohorts, and the assessment of outcome. Each study can be given a maximum of one star for each element within the sample selection process and the outcome and a maximum of two stars can be given for the comparability section. The sample selection section evaluated the: (1) representativeness of the exposed cohort (representative of populations presenting with acute LBP without red flags and exposed to eMRI scanning), (2) selection of non-exposed cohort, (3) ascertainment of the exposure and (4) demonstration that the outcome was not present at the start of the study. The comparability section evaluated: (1) whether a study adjusted for the most important factors deliberately and (2) whether a study adjusted for other important risk factors. The outcome section evaluated: (1) the method used to assess the outcome, (2) whether the follow-up period was long enough for outcomes to occur and (3) loss to follow up rate. To summarize the risk of bias in each study, we converted the Newcastle-Ottawa scales to the Agency for Healthcare Research and Quality (AHRQ) standards using the following recommended thresholds [28]: (1) good quality (3 or 4 stars in selection domain and 1 or 2 stars in comparability domain and 2 or 3 stars in outcome/exposure domain); (2) fair quality (2 stars in selection domain and 1 or 2 stars in comparability domain and 2 or 3 stars in outcome/exposure domain); (3) poor quality (0 or 1 star in selection domain OR 0 stars in comparability domain OR 0 or 1 stars in outcome/exposure domain).

#### Data extraction

The following data were extracted: study aim, source of funding, source of data, methods of data collection, study design, setting, follow up duration, population, number of participants, demographics, definition of LBP, definition of eMRI, definition of LOD, outcomes of association between eMRI and LOD, strengths and limitations, and conclusion. Data extraction was undertaken independently by two reviewers. Any disagreements were resolved by unanimity after involving a third reviewer. Where there was a conflict of interest or potential reviewer bias, the reviewer in question was not involved in the data extraction. Contacting authors for any missing data was considered. However, all required data was presented in the included papers.

#### Data analysis

Meta-analysis was considered but owing to between-study heterogeneity in measures of association between eMRI and LOD reported in included studies, formal pooling of the results was not feasible. Therefore, a narrative synthesis of results was conducted. Narrative synthesis was presented as reported in the original study and no additional analysis/synthesis were conducted.

## Results

### Study selection

Search strategies identified 354 records (Medline 93, EMBASE 187, CINAHL 74). After the removal of duplicates, 262 reports remained for the title and abstract screening. A total of 248 reports were excluded based on title and abstract. After the full-text screening, a further 9 studies did not meet the review inclusion criteria and were excluded (Fig. 1) [10, 29–36]. Three reports (Shraim et al., 2015, 2017, and 2019) [6, 7, 13] used the same sample at the same time in the same settings but addressing different objectives. Therefore, a total of 7 studies were included in the quality assessment stage of this systematic review (Fig. 1) [6, 7, 10–13, 37].

### Study characteristics

The characteristics of the included studies are presented in Table 1. All included studies were conducted in the United States (US) and used workers’ compensation (WC) administrative databases. Six studies used a retrospective cohort study design [6, 7, 11–13, 37], and one study used a prospective cohort study design [10]. Three studies by Shraim and colleagues [6, 7, 13] used the same sample to examine the relationship between different individual-level variables (including eMRI) with neighborhood and state-level variables and LOD in LBP cases (see Table 3 for list of variables included in each study). The sample size ranged from 98 to 59,360 with a total number of 64,232 LBP cases in all studies. The proportions of males ranged from 69 to 73%. The mean age of participants ranged between 39.4 and 41.4 years [6, 7, 11, 13, 37]. The median age was 34 years in one study [12], and one study included individuals aged 16–61 years but no summary measure of age was provided [10]. All studies included cases with uncomplicated LBP identified using ICD-9 codes [6, 7, 11, 13, 37], nature of injury codes [10], or combinations of body part and nature of injury codes [11]. eMRI was defined as lumbar MRI within 30 days [6, 7, 11, 13, 37] or 6 weeks of seeking medical care [10]. The LOD was defined as the total number of days of continuous paid indemnity (lost wage replacement for temporary total or temporary partial lost days) and truncated at either 1-year [6, 7, 11, 13, 37] or 2-year of follow-up periods [11, 37].

**Table 1:** Characteristics of included studies

**Study**	**Design**	**Data source & setting**	**Sample size**	**Population**
Shraim 2019 [13]	Retrospective cohort	A private workers’ compensation administrative and medical billing database, included cases from 49 states	59,360, all cases from 2002 to 2008	Persons aged 18–65 years, 69% males, mean age = 39.4 years (standard deviation (SD) 10.8)
Shraim 2017 [7]	Retrospective cohort	A private workers’ compensation administrative and medical billing database, included cases from 49 states	59,360, all cases from 2002 to 2008	Persons aged 18–65 years, 69% males, mean age = 39.4 years (standard deviation (SD) 10.8)
Shraim 2015 [6]	Retrospective cohort	A private workers’ compensation administrative and medical billing database, included cases from 49 states	59,360, all cases from 2002 to 2008	Persons aged 18–65 years, 69% males, mean age = 39.4 years (standard deviation (SD) 10.8)
Webster 2013 [11]	Retrospective cohort	A private workers’ compensation administrative and medical billing database, and included cases from 45 states	555, random sample of cases in 2006	Mean age = 41 years; 73.3% males
Graves 2012 [10]	Prospective cohort	Washington workers’ compensation program administrative and medical billing database plus telephone interviews	955, all cases between July 2002 and April 2004	Persons older than 18 years, 73.3% males
Webster 2010 [37]	Retrospective cohort	A private workers’ compensation administrative and medical billing database, and included cases from 45 states	3264, all cases in 2006	Mean age = 41.4 years, 69.7% males
Mahmud 2000 [12]	Retrospective cohort	A private workers’ compensation administrative database and included cases from 44 states and District of Columbia	98, random sample of cases Between June 1995 and August 1995	Persons aged 16–61 years, median age = 34 years, 71.4% males
**Study**	**LBP Definition**	**Exclusion criteria**	**eMRI definition**	**Work disability definition**
Shraim 2019 [13]	Uncomplicated LBP cases were identified using ICD-9 codes during the first 15 days of seeking medical care	Complicated LBP cases with severe injuries, multiple traumas, or significant non-injury diagnosis	Lumbar MRI within first 30 days of seeking medical care	Number of days of continuous paid indemnity (lost wage replacement for temporary total or temporary partial lost days) and truncated at the end of the 1-year follow-up period
Shraim 2017 [7]	Uncomplicated LBP cases were identified using ICD-9 codes during the first 15 days of seeking medical care	Complicated LBP cases with severe injuries, multiple traumas, or significant non-injury diagnosis	Lumbar MRI within first 30 days of seeking medical care	Number of days of continuous paid indemnity (lost wage replacement for temporary total or temporary partial lost days) and truncated at the end of the 1-year follow-up period
Shraim 2015 [6]	Uncomplicated LBP cases were identified using ICD-9 codes during the first 15 days of seeking medical care	Complicated LBP cases with severe injuries, multiple traumas, or significant non-injury diagnosis	Lumbar MRI within first 30 days of seeking medical care	Number of days of continuous paid indemnity (lost wage replacement for temporary total or temporary partial lost days) and truncated at the end of the 1-year follow-up period
Webster 2013 [11]	Uncomplicated LBP cases or radiculopathy were identified using ICD-9 codes	Complicated LBP cases with severe injuries, multiple traumas, chronic or recurrent LBP or prior lumbar surgery, those with limited clinical information, significant non-injury diagnosis, receipt of nonlumbar MRI, or those with limited clinical information	Lumbar MRI within first 30 days of seeking medical care	Number of days of continuous paid indemnity (lost wage replacement for temporary total or temporary partial lost days) and truncated at the end of the 2-year follow-up period
Graves 2012 [10]	Acute LBP was classified according to nature of injury (mild or major sprain/strain)	Cases with incomplete follow-up interviews, language limitations, severe injury, lack of information on the severity of the injury and/or disability compensation	Lumbar MRI within 6 weeks of seeking medical care	Same as Shraim 2019
Webster 2010 [37]	Uncomplicated LBP cases were identified using ICD-9 codes during the first month of seeking medical care	Cases with fractures, concurrent injuries within the first month from the date of injury, those with a work-related LBP claim in the prior year, those who had lumbar surgery before MRI and those with lump-sum settlements	Lumbar MRI within first 30 days of seeking medical care	Same as Webster 2013
Mahmud 2000 [12]	Acute LBP was classified according to nature of injury (mild or major sprain/strain)	Complicated LBP cases with severe injuries, multiple traumas, chronic or recurrent LBP, or concurrent workers’ compensation claim	Lumbar MRI within first 30 days of seeking medical care	Number of days of continuous paid indemnity (lost wage replacement for temporary total or temporary) followed by more than 7-day period without indemnity payments, and truncated at the end of the 1-year follow-up period

*Abbreviations: WC* Workers’ compensation, *SD* Standard deviation, *ICD* International classification of disease, *LBP* Low back pain, *eMRI* Early magnetic resonance imaging, *LOD* Length of disability

### Quality assessment

None of the studies examined in the quality assessment stage were excluded. All included studies were of good methodological quality. Six studies scored nine stars [6, 7, 11–13, 37] and one scored eight stars due to 30% loss to follow up [10] (Table 2). The score given to the representativeness of the exposed cohort was based on the study population which may differ in characteristics of the general population. One of the current review authors (MS) is an author in three of the included studies, therefore, MS was not involved in the quality assessment and any subsequent data extraction of Shraim and colleagues’ studies [6, 7, 13]. A total of 2 out of the 7 included studies had reviewer disagreement in relation to the outcome score of Newcastle-Ottawa scale. This disagreement was resolved by referring the two studies in question to a third reviewer (BA).

**Table 2:** Quality assessment of studies using the Newcastle-Ottawa scale

Study	Selection	Comparability	Outcome	Total Quality Score
Shraim 2019 [13]	****	**	***	9
Shraim 2017 [7]	****	**	***	9
Shraim 2015 [6]	****	**	***	9
Webster 2013 [11]	****	**	***	9
Graves 2012 [10]	****	**	**	8
Webster 2010 [37]	****	**	***	9
Mahmud 2000 [12]	****	**	***	9

### The association between eMRI and LOD

All included studies investigated the association between eMRI and LOD. The studies used multivariable analyses and adjusted for potential confounders. The main variables that all studies consistently adjusted for were age and gender (Table 3). Five studies followed up the patients for a duration of 1 year [6, 7, 10, 12, 13], and reported unadjusted mean (standard deviation (SD)) of LOD of 142.2 (125.0), 142.2 (125.0), 163.5 (144.6), 115 (not reported), and 142.2 (125.0) days in the eMRI group compared to 79.6 (105.1), 79.6 (105.1), 42.6 (86.6), 13 (not reported), and 79.6 (105.1) days in the no eMRI group, respectively. One of these studies did not report the SD for the LOD [12]. Two studies [11, 37] followed up patients for a duration of 2 years and reported unadjusted means of LOD of 128.5 (95% CI 128.5, 201.5) and 133.6 (95% CI 120.0,146.7) days in the eMRI groups compared to 44.4 (95% CI 37.5, 51.4) and 22.9 (95% CI 19.5, 26.2) days in the no eMRI groups, respectively. Two studies reported unadjusted means of LOD for LBP patients with radiculopathy of 184.0 (95% CI 154.8, 213.2) and 215.3 (SD = 127.5) days in the eMRI group compared to 50.0 (95% CI 38.0, 61.9) and 121.3 (SD = 142.6) days in the none-eMRI group, respectively (see Table 3) [10, 11]. Three studies reported adjusted geometric mean of LOD of 39.6 (95% CI 36.0, 43.6), 37.7 (95% CI 33.2, 42.2), and 37.8 (95% CI 33.9, 41.9) days in the eMRI groups compared to 25.9 (95% CI 23.0, 29.1), 24.4 (95% CI 21.4, 28.0), and 28.4 (95% CI 25.4, 31.7) days in the no eMRI groups at 1-year follow up, respectively [6, 7, 13]. These three studies reported that the eMRI groups had a higher adjusted mean LOD than the no eMRI groups by 9.4 days (95% CI 8.5, 10.2) [13], 13.3 days (95% CI 11.8, 14.8) [7], and 13.7 days (95% CI 13.0, 14.5) [6]. Four studies reported the hazard ratio (HR) as a measure of association between eMRI and work disability. Three studies reported that the eMRI groups had a higher HR of increased LOD than the no eMRI groups by 1.75 (95% CI 1.23, 2.50) [10], 2.91 (95% CI 1.45, 5.84) [12], and 3.13 (95% CI 2.33, 4.17) [11]. Two studies [10, 11] reported that eMRI groups with LBP and radiculopathy had a higher HR of increased LOD than the no eMRI groups with LBP and radiculopathy by 2.08 (95% CI 1.67, 2.63) [10] and 3.57 (95% CI 2.33, 5.56) [11]. One study controlled for potential MRI indication bias using the propensity of belonging to the eMRI group, computed based on demographic and severity indicators with adjustment for potential residual confounding of covariates [37]. This study reported that low-propensity eMRI subgroup had a higher HR of increased LOD than the low-propensity no eMRI subgroup and high-propensity no eMRI subgroup by 3.0 (95% CI 2.6, 3.4) and 2.9 (95% CI 2.3, 3.5), respectively [37].

**Table 3:** The relationship between eMRI and length of disability among low back pain cases

Study	Follow-up duration (years)	Study aim	Association between eMRI and LOD	Variables adjusted for in multivariable analysis	Study conclusion
Shraim 2019 [13]	1	“To explore whether the known risk of increased LOD associated with eMRI scanning not adherent to guidelines for occupational LBP varies according to patient and area-level characteristics, and the potential reasons for any observed variations.”	The eMRI group had a longer mean LOD by 9.4 days (95% CI^1^ 8.5, 10.2) compared to the no eMRI group.	Age, gender, tenure, average weekly wage, industry, injury severity, morphine equivalent amount in first 15 days of seeking medical care, lumbar spine surgery, litigation status, neighborhood median household income, state physician density, state orthopedic surgeons’ density, state MRI facility rate, and state workers’ compensation policies on wage replacement rate, waiting period, retroactive period, treating provider choice and change.	“State WC policies regulating selection of healthcare provider and structural factors affecting quality of medical care modify the impact of eMRI not adherent to guidelines. Targeted healthcare and work disability prevention interventions may improve work disability outcomes in patients with occupational LBP.”
Shraim 2017 [7]	1	“To determine if SE characteristics of claimants’ geographic context were associated with WC benefits including intensity of medical care (as reflected by medical expenses) and length of time absent from work for acute uncomplicated LBP, after controlling for individual and state characteristics.”	The eMRI group had a longer mean LOD by 13.3 days (95% CI 11.8, 14.8) than the no eMRI group.	Age, gender, average weekly wage, industry type, injury severity, early opioid/100 mg MEA in first 15 days, lumbar spine surgery, claim litigation status, neighborhood median household income, and state variables (state WC policies (wage replacement rate, waiting period, retroactive period, treating provider choice status, treating provider change status, state medical fee schedule status) unemployment rate, MRI facility rate, state physician density, orthopedic surgeons’ density).	“Regional SE disparities in medical costs and LOD occur even when health insurance, health care availability, and indemnity benefits are similar. Results suggest opportunities to improve care and disability outcomes through targeted health care and disability interventions.”
Shraim 2015 [6]	1	“To examine the impact of state WC policies regarding wage replacement and medical benefits on medical costs and LOD in workers with LBP.”	The eMRI group had a longer mean LOD by 13.7 days (95% CI 13.0, 14.5) than the no eMRI group.	Age, gender, average weekly wage, industry type, injury severity, early opioid/100 mg MEA in first 15 days, lumbar spine surgery, claim litigation status, work-live in same state status, neighborhood variables (median household income, rural population percentage, white population percentage, education attainment (percentage less than some college education), and state variables (state WC policies, wage replacement rate, waiting period, retroactive period, treating provider choice status, treating provider change status, state medical fee schedule status) percentage of population below 100% poverty, Gini coefficient percentage, unemployment rate, disabled workers receiving social security disability insurance).	“WC policies about wage replacement and medical treatment appear to be associated with WC LBP outcomes and might represent opportunities to improve LOD and reduce medical costs in occupational LBP.”
Webster 2013 [11]	2	“To determine the effect of early (receipt ≤30 d post-onset) magnetic resonance imaging (MRI) on disability and medical cost outcomes in patients with acute, disabling, work-related low back pain (LBP) with and without radiculopathy.”	At two-year follow-up, the mean LOD was 165 days (95% CI 128.5, 201.5) and 44.4 days (95% CI 37.5, 51.4) in the eMRI group and the no eMRI group, respectively. The eMRI group had a lower hazard ratio for going off disability by 0.32 (95% CI 0.24, 0.43) compared to the no eMRI group.	Age, sex, job tenure, jurisdiction state, morphine equivalent amount in first 15 days, time to first lumbar MRI, and average weekly medical costs pre-MRI.	“Early MRI without indication has a strong iatrogenic effect in acute LBP, regardless of radiculopathy status. Providers and patients should be made aware that when early MRI is not indicated, it provides no benefits, and worse outcomes are likely.”
Graves 2012 [10]	1	“To evaluate the association of early imaging and health and disability status 1 year following acute low back injury, among a population-based sample of Washington State workers’ compensation claimants.”	At one-year follow-up, the mean LOD was 163.5 days (standard deviation (SD) = 144.6) and 42.6 days (SD = 86.6) in the eMRI group and the no eMRI group, respectively. The eMRI group had a higher hazard ratio for work disability by 2.03 (95% CI 1.33, 3.11) than the no eMRI group.	Age, sex, race/ethnicity, education level, household income, marital status, body mass index, health status in year before injury, health status at baseline interview, baseline pain, Roland-Morris disability questionnaire scores, pain intensity, quality of life (role physical, physical functioning, and mental health scores), catastrophizing, work-fear avoidance, offered job accommodation for disability, previous LBP status, job satisfaction, industry, physical demands at work, and type of first medical visit.	“Among workers with LBP, early MRI is not associated with better health outcomes and is associated with increased likelihood of disability and its duration. These associations warrant further testing in a randomized controlled trial. Our findings suggest that adherence to evidence-based guidelines is an important factor in ensuring that workers receive the highest quality care for occupational injuries.”
Webster 2010 [37]	2	“To examine early magnetic resonance imaging (MRI) utilization for workers compensation cases with acute, disabling low backpain and further, to examine low or high propensity to undergo early MRI with disability duration, medical costs, and surgery.”	At two-year follow-up, the mean LOD was 133.6 days (95% CI 120.5, 146.7) and 22.9 days (95% CI 19.5, 26.2) in the eMRI group and the no eMRI group, respectively. To control for potential MRI indication bias, the propensity of belonging to the eMRI group was computed on the basis of demographic and severity indicators with adjustment for potential residual confounding of covariates. As compared to the eMRI (low propensity): The no MRI (low propensity) group had a hazard ratio of 3.0 (95% CI 2.6, 3.4) to go off disability. The no MRI (high propensity) group had a hazard ratio of 2.9 (95% CI 2.3, 3.5) to go off disability	Age, gender, job tenure, pre-MRI medical costs and disability, days to first MRI, early opioid use and MEA dose, maximum severity pre-MRI, and state of residence	“The majority of cases had no early MRI indications. Results suggest that iatrogenic effects of early MRI are worse disability and increased medical costs and surgery, unrelated to severity.”
Mahmud 2000 [12]	1	“To determine whether health care utilization and the physician’s initial management of work-related LBP were associated with disability duration.”	The no eMRI group had a higher risk ratio for going off disability by 2.91 (95% CI 1.45, 5.84) compared to the eMRI group.	Gender, physical job, demand, main care provider (primary care or specialist), main care setting (private clinic, urgent care or others) specialist referral, number of physician visit, multiple MD care status, clinical severity at initial presentation, diagnostic imaging status (x-ray, CT, and ultrasound status), opioid prescription status, and other therapeutic intervention (bed rest, back stretch, and back exercise status)	“The nature of the association between these initial clinical management aspects and LBP disability duration merits further exploration.”

*Abbreviations: WC* Workers’ compensation, *LBP* Low back pain, *eMRI* Early magnetic resonance imaging, *LOD* Length of disability, *SE* Socioeconomic, *CI* Confidence interval, *MEA* Morphine equivalent amount

## Discussion

This systematic review examined the relationship between eMRI for LBP without red flags and LOD. All included studies showed that subjects who received eMRI for LBP had an increased LOD than those who did not receive eMRI. The findings of our systematic review are consistent with the findings of a previous systematic review of two studies which concluded that patients with acute non-specific LBP who received MRI had a higher LOD as compared to the no MRI group [25]. The current systematic review included 7 studies from 5 unique study populations and added further evidence that eMRI is associated with increased LOD in patients with LBP without red flags even after accounting for several factors associated with LOD in this population. The three studies by Shraim and colleagues used the same study population and found that eMRI was associated with increased LOD in patients presenting with acute LBP without red flags after accounting for neighborhood socio-economic characteristics and state-level variables, including WC policy characteristics [6, 7, 13]. One study by Graves and colleagues also showed that eMRI was associated with increased LOD in patients with LBP without red flags after accounting for baseline functional disability, pain severity, quality of life, catastrophizing, work-fear avoidance, job accommodation, previous LBP status, job satisfaction, industry, physical demands at work, and type of first medical visit [10].

Despite recommendations of clinical practice guidelines against eMRI scanning for acute LBP without red flags, significant proportions of patient with LBP receive eMRI [10–13, 37]. The exact reasons for this are not clear. Previous studies hypothesized that lack of adherence to clinical guidelines could be explained by several factors, including patient’s demand for diagnostic imaging, patient reassurance by diagnostic findings, concerns about litigation especially in WC settings, physicians’ inadequate awareness about the natural history of acute LBP, and inertia of previous experience, or outcome expectancy [11, 37–39].

This review used a comprehensive search strategy and searched key bibliographical databases and the grey literature to identify relevant studies. The included studies consisted of large samples of LBP cases and used WC administrative data which captures complete information on medical bills, treatment, interventions, and duration of work disability.

This review has some limitations that should be noted. *First*, the current review included a small number of studies (7 studies from 5 study populations). *Second,* the included studies in this review used WC databases as the primary source of data. This data does not provide information on some predictors of LOD, such as level of functional disability, work accommodation, nature of job, fear-avoidance, and other comorbidities, including psychiatric conditions. However, this is unlikely to influence the findings unless the distribution of those predictors differs significantly between the eMRI and no MRI groups. In addition, the study by Graves et al., found that eMRI group had an increased HR of LOD than the eMRI group even after controlling for baseline pain, Roland-Morris disability questionnaire scores, pain intensity, quality of life (role physical, physical functioning, and mental health scores), catastrophizing, work-fear avoidance, offered job accommodation for disability, previous LBP status, job satisfaction, industry, physical demands at work, and type of first medical visit [10]. *Third*, the included studies measured LOD using wage replacement data. This may underestimate the observed association between eMRI and increased LOD because termination of wage replacement does not necessarily translate to complete recovery or return to work. *Fourth*, all included studies were conducted in the US, which may limit the generalizability of the findings to other countries that have different healthcare systems. However, these studies have good methodological quality and reported consistent findings related to the review question. *Fifth*, formal pooling of the results using meta-analysis was not feasible owing to between-study heterogeneity.

More research is needed to uncover the exact reasons for ordering the non-indicated eMRI for acute LBP without red flags. This information is useful for developing interventions and strategies to improve adherence to clinical guidelines’ recommendations about the management of patients presenting with acute LBP.

## Conclusions

eMRI is associated with an increased LOD in patients with acute LBP without red flags. Further research is needed to fully understand the reasons for the use of non-indicated eMRI for patients presenting with LBP. Developing healthcare interventions to enhance adherence to clinical guidelines may improve disability outcomes among patients with LBP.

## Supplementary Information

**Additional file 1.** Prisma 2020 Checklist.

**Additional file 2.** Full search strategy.

**Additional file 3:.** Newcastle-Ottawa quality assessment scale for cohort studies.

## Data Availability

No new data were created or analyzed in this study. Data sharing is not applicable to this article.
