# Supplementary material for: The association between early MRI and length of disability in acute lower back pain: a systematic review and narrative synthesis
Source: BMC Musculoskelet Disord. 2021 Nov 24;22:983. doi: 10.1186/s12891-021-04863-9 (PMC8614033; doi:10.1186/s12891-021-04863-9)
Supplement: Supplementary file 2 — Additional file 2. Full search strategy. [file 12891_2021_4863_MOESM2_ESM.docx]

**Supplemental file S2** Full search strategy

|  | **Concept** | | |
| --- | --- | --- | --- |
| **Database** | **MRI** | **Low Back Pain** | **Work Disability** |
| MEDLINE | exp Magnetic Resonance Imaging/  “Magnetic Resonance Imaging”  “nuclear magnetic resonance”  MRI  NMR | exp low back pain/  exp sciatica/  sciatica  “low* back pain”  “low* back injury”  lumbago  “lumbosacral pain”  “lumbar pain”  radiculopathy  “radicular pain” | exp return to Work/  Insurance, Disability/  Sick leave/  Absenteeism/  work adj3 disability  work adj3 absence  work adj3 absenteeism  work adj3 incapacity  sick adj3 leave  sickness adj3 leave  sick adj3 period  sickness adj3 period  sick adj3 duration  sickness adj3 duration  disability adj3 duration  disability adj3 period  “time lost” adj3 work  “time loss” adj3 work  return* adj3 work  “back to” adj3 work  worktime adj3 loss  “work time” adj3 loss  workday* adj3 loss  worktime adj3 lost  “work time” adj3 lost  workday* adj3 lost |
| EMBASE | nuclear magnetic resonance imaging/  “Magnetic Resonance Imaging”  “nuclear magnetic resonance”  MRI  NMR | exp low back pain/  exp ischialgia/  sciatica  “low* back pain”  “low* back injury”  lumbago  “lumbosacral pain”  “lumbar pain”  radiculopathy  “radicular pain” | work disability/  work resumption/  return to work/  absenteeism/  work adj3 disability  work adj3 absence  work adj3 absenteeism  work adj3 incapacity  sick adj3 leave  sickness adj3 leave  sick adj3 period  sickness adj3 period  sick adj3 duration  sickness adj3 duration  disability adj3 duration  disability adj3 period  “time lost” adj3 work  “time loss” adj3 work  return* adj3 work  “back to” adj3 work  worktime adj3 loss  “work time” adj3 loss  workday* adj3 loss  worktime adj3 lost  “work time” adj3 lost  workday* adj3 lost |
| CINAHL | exp Magnetic Resonance Imaging/  “Magnetic Resonance Imaging”  “nuclear magnetic resonance”  MRI  NMR | exp Low Back Pain/  sciatica  “low* back pain”  “low* back injury”  lumbago  “lumbosacral pain”  “lumbar pain”  radiculopathy  “radicular pain” | Insurance, Disability/  Absenteeism/  Sick Leave/  Employee, Disabled/  work adj3 disability  work adj3 absence  work adj3 absenteeism  work adj3 incapacity  sick adj3 leave  sickness adj3 leave  sick adj3 period  sickness adj3 period  sick adj3 duration  sickness adj3 duration  disability adj3 duration  disability adj3 period  “time lost” adj3 work  “time loss” adj3 work  return* adj3 work  “back to” adj3 work  worktime adj3 loss  “work time” adj3 loss  workday* adj3 loss  worktime adj3 lost  “work time” adj3 lost  workday* adj3 lost |
